# Nordic consensus document on Evolut FX+ transcatheter aortic valve implantation: optimizing index implantation and longer-term outcomes

**DOI:** 10.3389/fcvm.2025.1682714

**Published:** 2025-11-10

**Authors:** Arif A. Khokhar, Mikko Savontaus, Ahmed Al-Ani, Christian Juhl Terkselsen, Gintautas Bieliauskas, Annemieke van der Heijden, Azad Alhasan Alhaj Amin, Stefan James, Kristoffer Russell, Ole De Backer

**Affiliations:** 1 Rigshospitalet, Copenhagen University Hospital, Copenhagen, Denmark; 2 Heart Center, Turku University Hospital, Turku, Finland; 3 Department of Cardiology, Ullevål Oslo University Hospital/Ullevål, Oslo, Norway; 4 Department of Cardiology, Aarhus University Hospital, Aarhus, Denmark; 5 Education & Training, Medtronic, Tolochenaz, Switzerland; 6 Department of Cardiology, Uppsala University Hospital, Uppsala, Sweden; 7 Department of Medical Sciences, Cardiology, Uppsala University, Uppsala, Sweden; 8 Department of Cardiology, Rikshospitalet, Oslo University Hospital, Oslo, Norway

**Keywords:** transcatheter aortic valve replacement, newest-generation technology, optimized implantation technique, commissural alignment, coronary access

## Abstract

Transcatheter aortic valve replacement (TAVI) is an established treatment strategy for patients with severe symptomatic aortic stenosis (AS). Multiple landmark randomized controlled trials have consistently demonstrated the safety, efficacy, and longer-term durability of the CoreValve/Evolut (Medtronic, MN, USA) transcatheter aortic valve (TAV) platform in treating severe AS. These findings have supported the expansion of TAVI to younger patients with longer life expectancy, in whom an optimized index valve implantation can significantly impact both acute procedural results and longer-term outcomes. In this technical narrative, we aim to describe how iterative changes in the latest-generation Evolut FX(+) TAV can be utilized to achieve an optimized index valve implantation.

## Introduction

Transcatheter aortic valve replacement (TAVI) is an established treatment strategy for patients with severe symptomatic aortic stenosis (AS) (1, 2). Multiple landmark randomized controlled trials have consistently demonstrated the safety and efficacy of the CoreValve/Evolut (Medtronic, MN, USA) transcatheter aortic valve (TAV) platform in treating severe AS (3–5). Long-term follow-up studies have confirmed stable valve hemodynamics and low rates of structural valve deterioration (SVD), comparing favorably with other surgical and transcatheter aortic bioprostheses (6–9). These findings have supported the expansion of TAVI to younger patients with longer life expectancy (10, 11). For this growing cohort of patients, the index valve implantation can significantly impact both acute procedural results and longer-term outcomes on valve durability, coronary access, and redo-TAVI feasibility (12–14). Therefore, achieving an optimal index valve implantation is an essential first step in establishing an effective lifetime management strategy for younger patients.

In this technical narrative, we aim to describe how iterative changes in the latest-generation Evolut FX(+) TAV can be utilized to achieve an optimized index valve implantation.

## Evolut FX and Evolut FX+ systems

The first-generation CoreValve TAV, which received CE marking in 2007, has since undergone multiple iterations, culminating in the latest-generation Evolut FX and Evolut FX+ systems (15). Early registry data have demonstrated promising results, with high technical success (99.1%) and low 30-day rates of mortality (1.3%), stroke (1.3%), major vascular complications (0.9%), and new pacemaker implantation (11.9%) (16).

**Figure 1:**
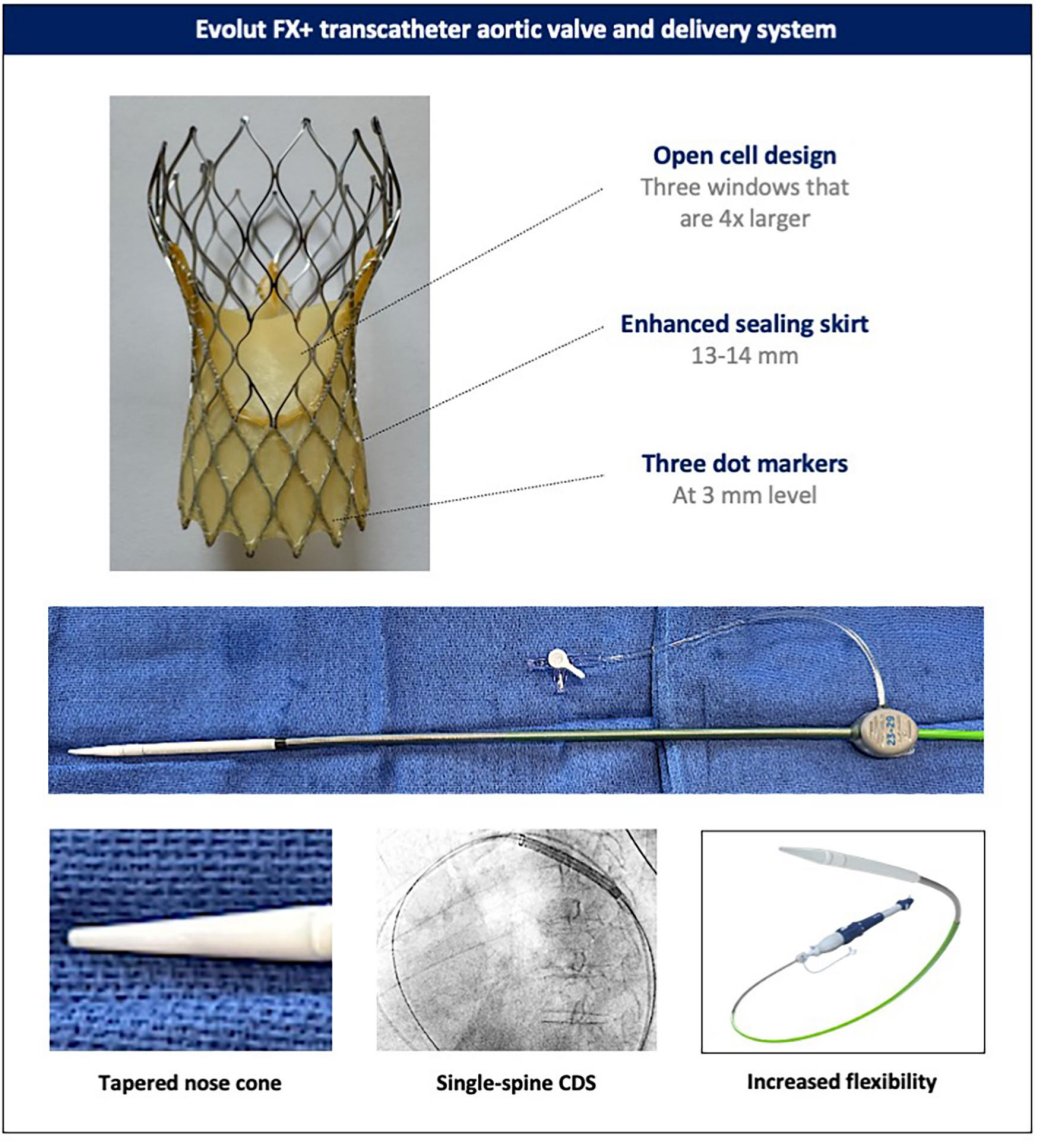
Evolut FX+ transcatheter aortic valve and delivery system. Novel design features of the latest-generation Evolut FX+ valve and its catheter delivery system (the catheter delivery system of the Evolut FX and FX+ valves). CDS, catheter delivery system.

Modifications have been made to both the catheter delivery system (CDS) and the valve compared with the Evolut PRO+ system (Figure 1). The CDS of the Evolut FX(+) has been redesigned to include a more tapered nose cone, a single rather than double spine to the shaft, and the addition of a stability layer, which altogether render the system more flexible and easier to track but with sufficient stability during valve deployment. The increased flexibility allows the CDS to be torqued in the descending aorta to achieve commissural alignment (see below). Moreover, three radiopaque dot markers have been incorporated 3 mm from the inflow of the Evolut FX(+) stent frame, in line with the commissural posts, to facilitate the assessment of implantation depth, co-axiality (or parallax), and commissural alignment. Altogether, these changes have led to improved implantation symmetry, consistently achieving a target implantation depth of 1–5 mm, with fewer recaptures compared to the Evolut PRO+ (16). Further improvements in both commissural alignment and subsequent coronary access have also been demonstrated. In the Evolut FX+ system, the additional three large diamond-shaped cells further facilitates coronary access, addressing one of the main challenges associated with tall-frame supra-annular valves (16–18).

**Figure 2:**
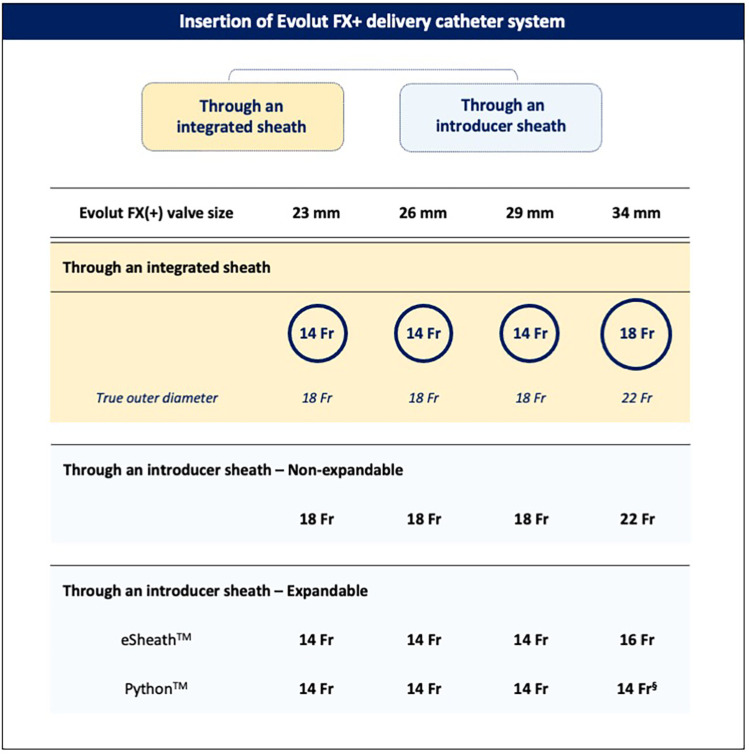
Insertion of the Evolut FX+ catheter delivery system. The Evolut FX(+) valve can be implanted using either the integrated delivery catheter system or an introducer sheath. ^§^Following pre-dilatation of the Python 14-Fr sheath with a 7- or 8-mm balloon.

The valve can be implanted using an integrated “in-line” sheath, which is 14 Fr-equivalent [true outer diameter (OD) 18 Fr] for the 23-, 26-, and 29-mm valves and 18 Fr-equivalent (true OD 22 Fr) for the 34-mm valve. When selecting a non-expandable introducer sheath, an 18-Fr sheath is required for the 23–29-mm valves and a 22-Fr sheath for the 34-mm valve. For expandable introducer sheaths, the 23–29-mm valves are compatible with the 14-Fr eSheath™ (Edwards Lifesciences, IR, USA) and 14-Fr iSleeve™ (Boston Scientific, MA, USA), while the 34-mm valve is compatible only with the 16-Fr eSheath (Figure 2).

In patients with hostile aortic arch anatomy, whether due to excessive calcification or heavy atheromatous plaque burden, a long introducer sheath such as the 65-cm-long DrySeal Flex (GORE, DE, USA), enables safe advancement and delivery of the TAV while minimizing the risk of aortic wall trauma and embolic material dislodgement. In addition, a long introducer sheath can help overcome challenges posed by extreme tortuosity and steep angulations of the aorta, facilitating stable and predictable valve deployment.

## Effective pre-dilatation

Prior to valve implantation, effective pre-dilatation should be considered to facilitate adequate stent frame expansion. The nitinol stent frame of the Evolut FX(+) is designed to adopt its intended shape and size upon release. When crimped into the delivery catheter, the stent frame stores energy. As the valve is unsheathed, this stored energy is translated into and generates the opening force of the valve, which pushes against the resistive force from the surrounding anatomy. Therefore, effective pre-dilatation modifies the leaflet calcifications and remodels the surrounding anatomy, thereby reducing the resistive forces acting against valve opening, improving favorable stent frame expansion and stability upon release, and reducing the need for multiple valve repositioning maneuvers and the risk for potential stent frame infolding.

**Figure 3:**
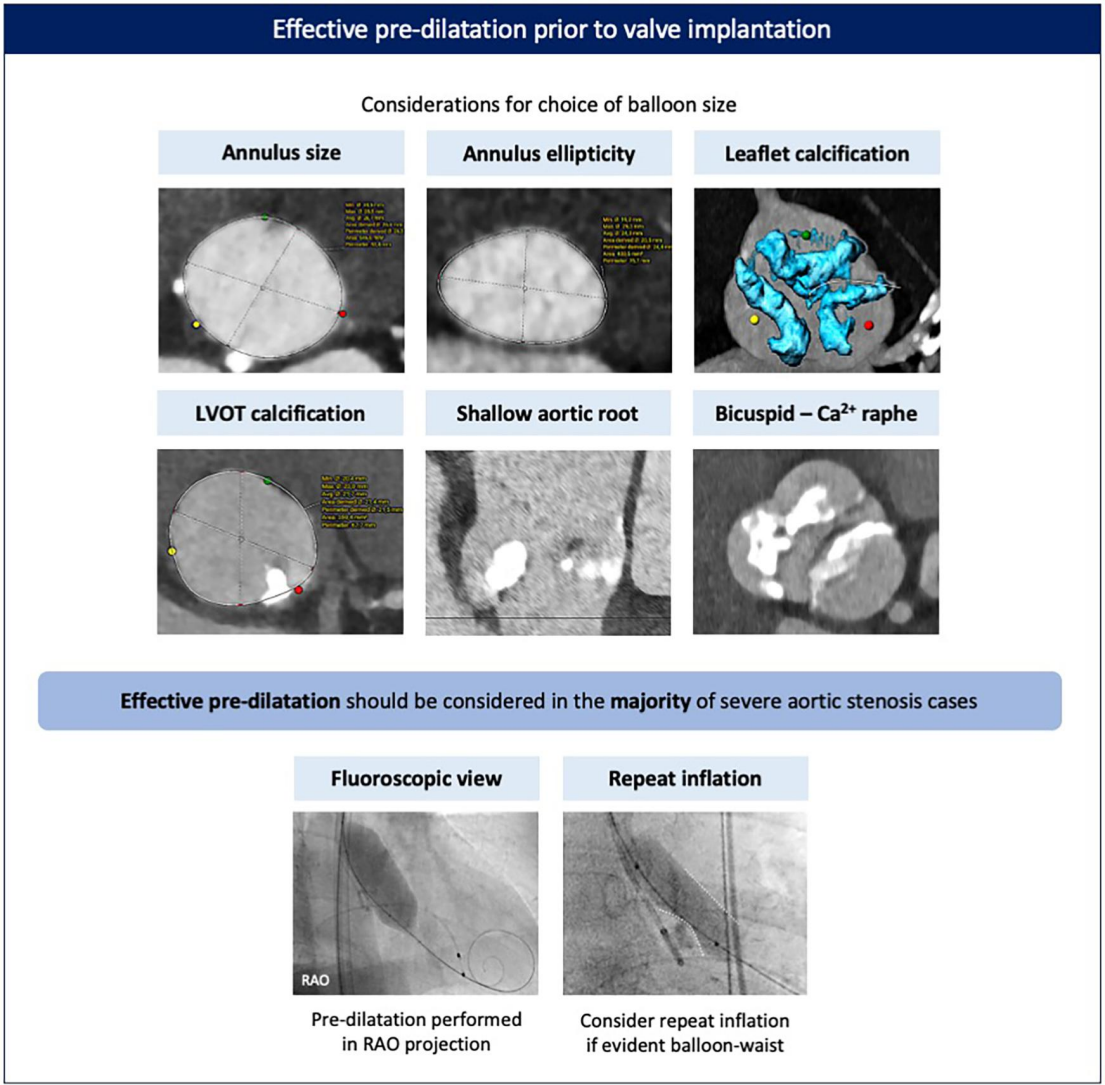
Effective pre-dilatation prior to valve implantation. Pre-dilatation should be considered for most cases prior to valve implantation, with balloon sizing based on the dimensions and phenotype of the aortic annulus and valve. Pre-dilatation is best performed in an RAO view to better appreciate balloon expansion and calcified leaflet modification. Repeat inflation may be advised in case of excessively calcified leaflets and/or if a significant waist on the balloon is evident. Ca, calcified; LVOT, left ventricular outflow tract; RAO, right anterior oblique.

The effectiveness of pre-dilatation can be influenced by anatomical factors (valve phenotype and calcification pattern) and procedural factors (balloon type, size, and inflation technique) (Figure 3). For pre-dilatation, a balloon 1–2 mm smaller than the perimeter-derived mean annulus diameter is usually recommended. In anatomies with a higher risk of mechanical aortic annulus or aortic root injury, such as cases with calcification at the aortic annulus/left ventricular outflow tract (LVOT), excessively calcified bicuspid valves with a long calcified raphe, or severe bulky leaflet calcification in combination with a shallow aortic root, a strategy of either balloon downsizing, guided by the minimal diameter, or using a semi-compliant balloon should be considered. Balloon pre-dilatation in a right anterior oblique (RAO) projection, which visualizes the minor axis of the aortic valve, can provide a more accurate estimate of balloon expansion and pre-dilatation adequacy. In cases of excessive leaflet calcification (e.g., bicuspid valves) or when a clear waist on the balloon is evident with pre-dilatation, a repeat balloon inflation or, in certain cases, pre-dilatation with a larger balloon may be considered if safe. Importantly, pre-dilatation should only be performed after checking and confirming appropriate Evolut valve loading to avoid the pitfall of hemodynamic collapse caused by severe aortic regurgitation after pre-dilatation (although this is very rare).

## Commissural alignment

Ensuring commissural alignment of an implanted TAV is important for preserving future coronary re-access, facilitating leaflet modification techniques, enabling redo-TAVI, and potentially benefitting long-term valve hemodynamics and durability (19–22). With the latest-generation Evolut FX+, which features three large diamond-shaped windows between the commissural posts (Figure 1), achieving commissural alignment is highly recommended to attain the maximum benefit of these larger cells for coronary access. The technique for commissural alignment with the Evolut FX(+) platform remains similar to previous generations (20). However, the addition of three commissural dot markers and a more responsive and flexible catheter delivery system has improved the success rate of achieving patient-specific commissural alignment from ∼80% with the previous-generation Evolut PRO+ to >96% with the Evolut FX(+) (16, 17).

**Figure 4:**
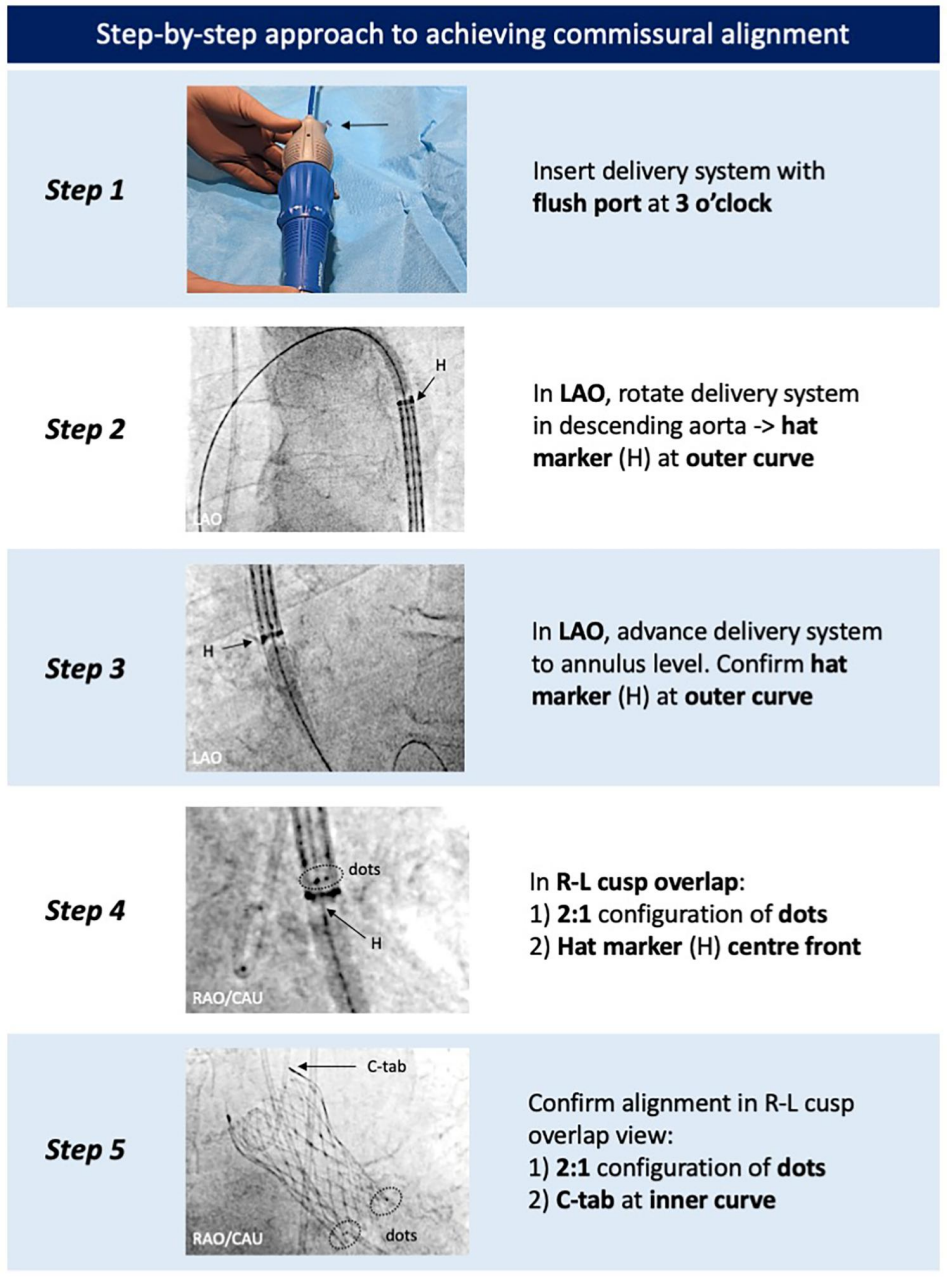
Step-by-step approach to achieve commissural alignment with the Evolut FX(+). Commissural alignment can be achieved by tracking the positions of the hat marker and specific radiopaque markers found on the catheter delivery system and Evolut FX(+) valve, respectively. LAO, left anterior oblique; R–L, right–left.

The first step is to insert the delivery catheter with the flush port pointing in the 3 o’clock direction. The valve is then advanced to the descending aorta, and under a 20°–30° left anterior oblique (LAO) projection, the catheter is gently torqued to position the hat marker on the outer curve. While maintaining the LAO projection, the valve is advanced toward the aortic annulus, ensuring the hat marker remains on the outer curve while crossing the aortic arch. Once at the aortic annulus, commissural alignment is confirmed using the patient-specific R–L cusp overlap view (obtained from the pre-TAVI CT), which places a native commissure on the right side of the screen. A 2:1 configuration of the marker dots, with one dot positioned on the right side of the screen, confirms that a TAV commissure is aligned with the native commissure. In addition, commissural alignment can be confirmed if the hat marker is positioned at the “center front” in the same R–L cusp overlap view, as the hat marker lies 90° to the Evolut FX(+) commissure. If commissural alignment has not been achieved and remains necessary, then it is recommended to withdraw the delivery system into the descending aorta to re-orient the hat marker along the outer curve in the LAO view, as the current-generation delivery system does not allow reliable intra-annular or ascending aortic rotation. Following Evolut FX(+) implantation, commissural alignment can easily be confirmed in the R–L cusp overlap view by confirming the 2:1 configuration of the marker dots and observing the C-tab position on the inner curve (Figure 4).

## Optimized implantation technique

**Figure 5:**
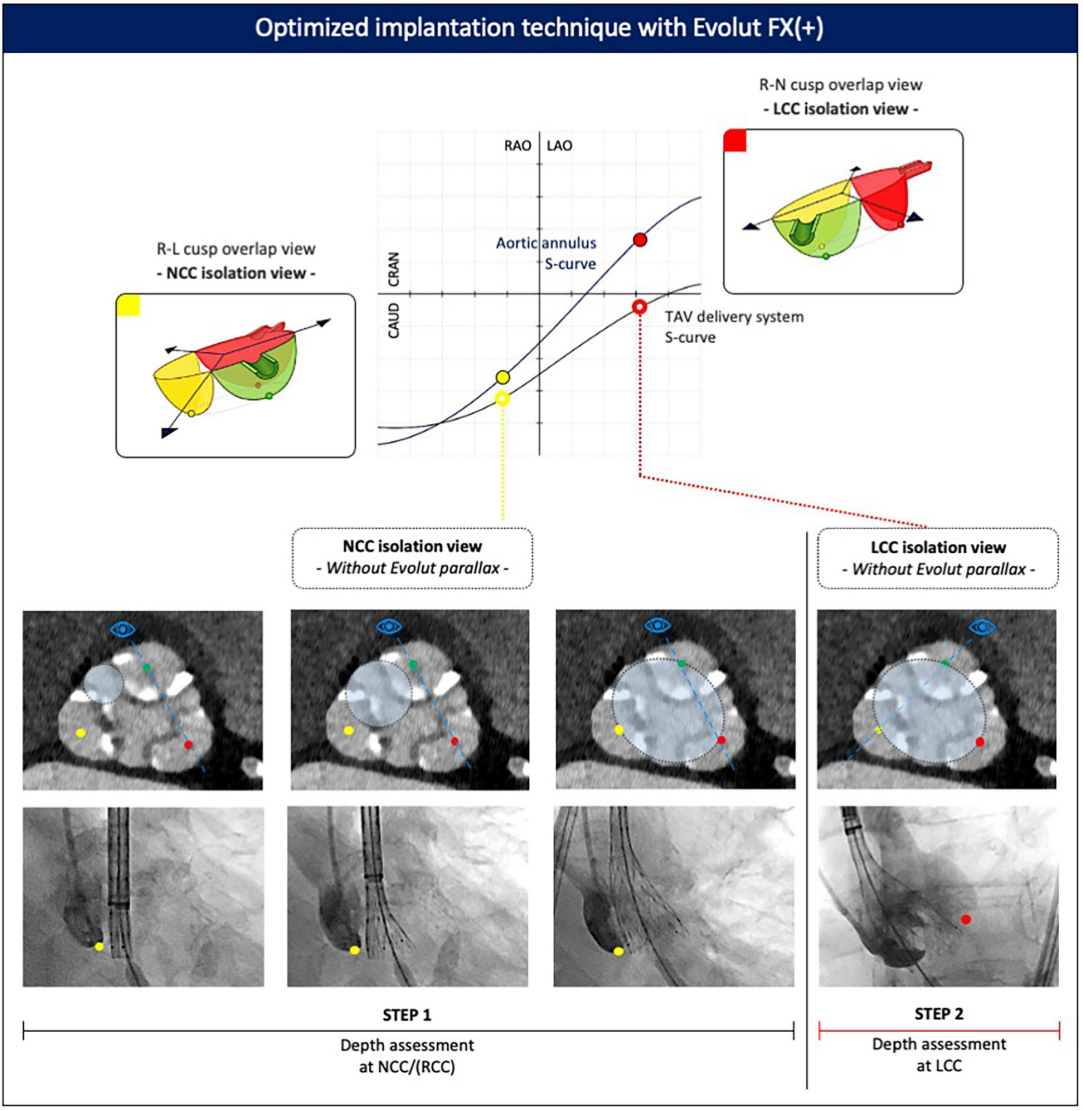
Optimized implantation technique with the Evolut FX(+). Valve implantation is commenced in the NCC isolation view. Assessment of the Evolut implant depth relative to the base of the NCC and LCC is then made in both the NCC and LCC isolation views, respectively, after removing parallax in the Evolut valve. CAU, caudal; CRA, cranial; LAO, left anterior oblique; LCC, left coronary cusp; NCC, non-coronary cusp; RAO, right anterior oblique; RCC, right coronary cusp; R–L, right–left; R–N, right–non.

The goal of optimized implantation is to achieve a maximally expanded Evolut TAV deployed at a target implant depth of 3 mm, with symmetrical positioning across all three aortic cusps, low transvalvular gradients, and minimal regurgitation. This can be achieved using a dedicated implantation technique, which requires an appreciation and understanding of the relative positions of the aortic cusps and the transcatheter heart valve during deployment (23) (Figure 5).

The first step is to obtain patient-specific C-arm projections corresponding to the non-coronary cusp (NCC) isolation view (approximately the R–L cusp overlap) and left coronary cusp (LCC) isolation view (approximately the R–N cusp overlap) from the pre-procedural CT scan. In particular, the degree of RAO or LAO is important, as it isolates or lateralizes the NCC or LCC, respectively. These two views are then used as the reference points to assess valve positioning and implantation depth during deployment.

Deployment of the Evolut FX(+) begins in the NCC isolation view, which offers several advantages. First, as the valve advances toward the aortic valve, it tracks along the path of the LV guidewire, which sits in the commissure between the NCC and RCC. In addition, the increased flexibility of the Evolut FX(+) delivery system enables the device to track along the outer curve of the aorta, making it more likely to cross the aortic valve in the commissure between the NCC and RCC. As a consequence, the NCC and RCC serve as the first contact points made between the Evolut FX(+) valve and the aortic annulus. Second, in the NCC isolation view, both the aortic annulus and delivery system are more likely to be seen in the “same plane” with minimal parallax between both structures, allowing for precise evaluation of the spatial relationship between these two structures and a more accurate assessment of implant depth at the NCC level, which is relevant given the location of the conduction system underneath the NCC/RCC. Third, positioning the pigtail catheter at the base of the NCC facilitates assessment of the Evolut FX(+) implant depth relative to the base of the NCC.

If there is evidence of parallax in the delivery system in the NCC isolation view (yellow full circle, Figure 5), the C-arm should be moved more caudally (or cranially) to eliminate the parallax. Applying a caudal (or cranial) tilt from the NCC isolation view has the effect of raising (or lowering) the position of the RCC relative to the LCC, but it will not affect the rotational position of the NCC relative to the RCC/LCC. This means that the base of the NCC can still be used as the reference point for assessing the implant depth of the Evolut FX(+) valve.

During the initial stages of valve unsheathing, there is sometimes a tendency for the valve to sink slightly down toward the LV. Initial valve opening in the NCC isolation view without Evolut parallax (yellow open circle, Figure 5) should be performed slowly, with small micro-adjustments of either the delivery system or the LV guidewire to maintain the Evolut FX(+) at the target implant depth, using the 3-mm dot markers on the delivery system as a reference. Initially, the valve can be unsheathed until node 3 after which controlled pacing is recommended to stabilize the system, particularly in high cardiac output states or in the presence of frequent ventricular ectopy. To minimize the hemodynamic impact of rapid pacing, this can be performed in a stepwise fashion, gradually increasing the pacing rate as the valve is progressively unsheathed, until the point of no recapture (80% deployment). At this stage, pacing can be stopped, as the leaflets are fully functioning, resulting in hemodynamic stability.

The next step is to check the implant depth and expansion of the Evolut TAV. If parallax in the Evolut FX(+) was introduced during valve deployment, it should be removed by adjusting the C-arm position (typically, by moving more caudally or into LAO). This maneuver is facilitated by aligning the three dot markers on the Evolut FX(+) frame. A contrast injection is used to confirm the implant depth of the Evolut valve relative to the NCC. Since the valve opens from the NCC–RCC commissure outward toward the LCC, the depth at the NCC can serve as a surrogate to estimate the depth at the RCC. Complete annular contact can be confirmed by assessing the amount of paravalvular leak, which may be significant if stent frame expansion is incomplete. Crowding of the stent frame struts at the inflow level may suggest severe regional under-expansion, and in such cases, it is also of utmost importance to rule out Evolut infolding, which appears as a longitudinal radiopaque line crossing the stent frame obliquely and can be confirmed using multiple fluoroscopic projections.

Next, the C-arm is rotated to the LCC isolation view, with cranial or caudal tilt applied to remove Evolut parallax (red open circle, Figure 5), and a contrast injection is repeated to determine the implant depth at the LCC. It is important to ensure a minimal 1–3 mm implantation depth is achieved relative to the base of the LCC. It is important to note that in the LCC isolation view, depth assessment of the Evolut FX(+) only relates to the LCC, and the depth at the NCC and RCC cannot be evaluated. Finally, in this projection, attention is given to the position of the delivery catheter within the aortic root, with a central position preferred to minimize the risk of the major valve canting upon final release. If the NCC or LCC isolation views require extreme angulations, a compromise can be achieved using a “near NCC or near LCC isolation view.” In this case, less extreme RAO or LAO angulations are applied while still removing parallax in the valve frame.

Following these evaluations in both fluoroscopic views (NCC and LCC isolation views), a decision is made to either continue with complete valve deployment or to partially or fully recapture and reposition the valve. This assessment should consider the following factors: (1) the hemodynamic status of the patient, (2) Evolut FX(+) stent frame expansion and annular contact (ensuring no valve infolding), (3) the extent of paravalvular leak, (4) the implant depth achieved at both the NCC/RCC and LCC (target ∼3 mm), (5) the position of the delivery catheter in the aortic root, which may influence valve stability and tilt upon release, and (6) the potential safety of a re-sheathing maneuver, which can be associated with increased peri-procedural risks.

If deemed suitable to proceed, the LV guidewire is typically slightly pulled back while maintaining contact with the LV for pacing if required. Controlled pacing can be applied to stabilize the valve during the final stages of deployment, which should be performed slowly and in a controlled fashion to allow the self-expandable valve to gradually make contact with the surrounding calcified leaflets and aortic root and maximize proper anchoring of the valve. Once the valve is fully deployed, attention should be given to ensure that both commissural tabs have been released from the delivery system, which can be confirmed using orthogonal fluoroscopic projections. If there is any doubt, or if one of the tabs remains attached, excessive pulling of the delivery system should be avoided, as this can lead to inadvertent migration and/or embolization. Applying gentle forward tension together with rotation, or a partial recapture followed by rotation and unsheathing, can help release the tab. Finally, the nose cone should be withdrawn carefully, ensuring that it does not contact or inadvertently pull the lower edge of the Evolut FX(+). Retracting the LV guidewire centralizes the nose cone within the valve, which facilitates its withdrawal in a safe and controlled manner.

## Effective post-dilatation

**Figure 6:**
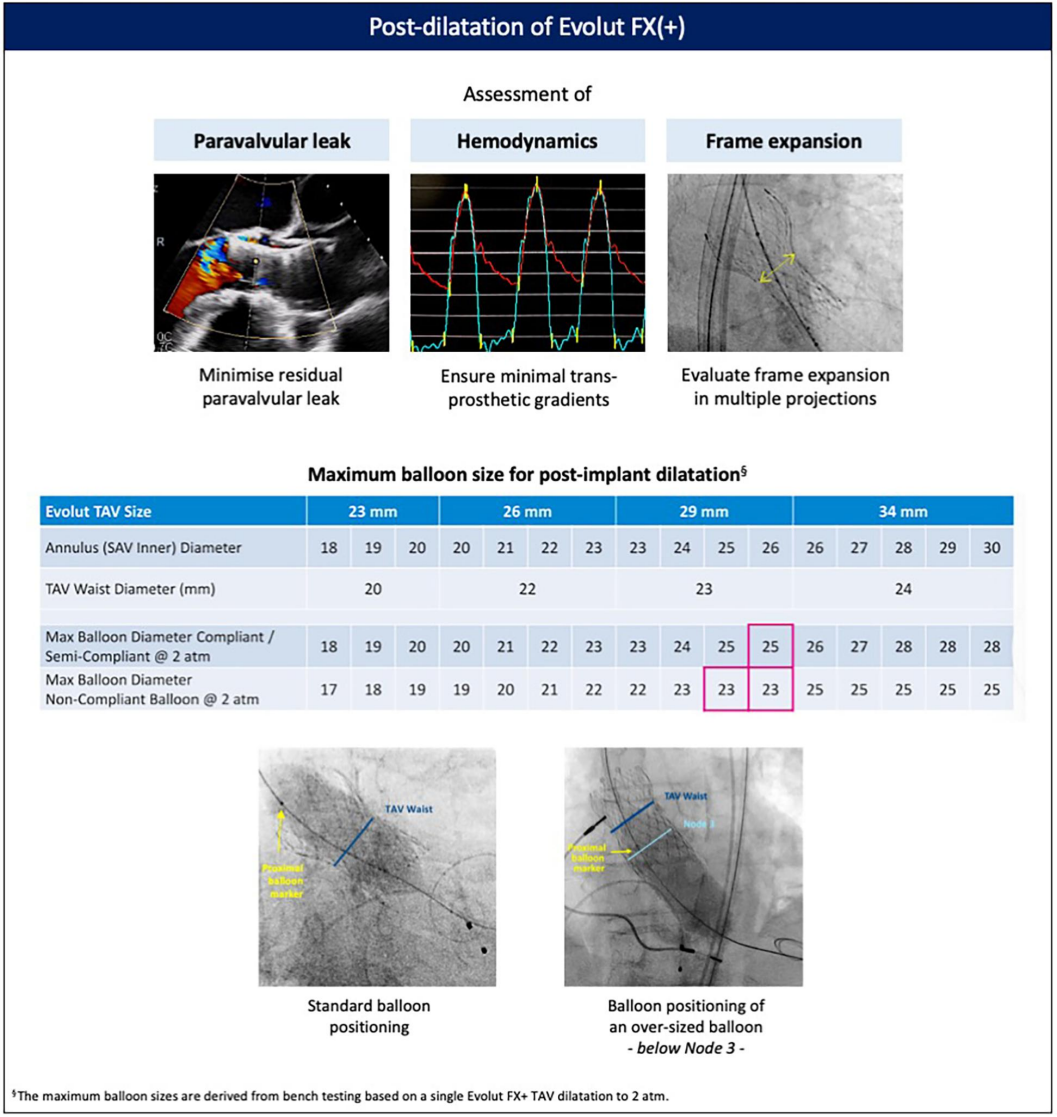
Post-dilatation of the Evolut FX(+). Post-dilatation is recommended to address significant paravalvular regurgitation, elevated transvalvular gradients, or stent frame under-expansion. Balloon sizing is based on the dimensions of the native aortic annulus and aortic root, the bulkiness of calcium at the level of the leaflets, annulus, and left ventricular outflow tract, and the waist of the implanted Evolut valve. TAV, transcatheter aortic valve.

Following Evolut FX(+) implantation, the decision to perform post-dilatation should be based on a multi-parametric assessment evaluating paravalvular regurgitation, valve hemodynamics (including the gradient between the aortic diastolic and left ventricular end-diastolic pressures), and stent frame expansion. The maximum recommended balloon size for post-dilatation is based on the waist diameter of the Evolut FX(+) valve and the type of balloon (non-compliant vs. semi-compliant) (Figure 6). Rapid pacing, usually at 180–220 bpm, is advised to ensure balloon stability and minimize the risk of valve embolization, and pacing should not be terminated before full balloon deflation. The balloon should be positioned such that its distal edge protrudes no more than 4 mm below the inflow of the stent frame to prevent excessive protrusion into the LVOT, which can compromise the conduction system. If, however, standard post-dilatation is insufficient due to significant residual paravalvular leak, a larger semi-compliant balloon can be used. In such rare cases, the balloon should be positioned with the proximal marker below the level of node 3 to expand the stent inflow without damaging the Evolut leaflets. If post-dilatation is being performed to correct for stent frame under-expansion, this assessment is best made in a RAO fluoroscopic view, where stent frame under-expansion is best appreciated and corrected.

## Coronary access

As TAVI expands to younger populations, preserving future coronary re-access becomes increasingly important. The supra-annular location of the leaflets, tall commissural posts, and relatively smaller cell size of the previous-generation Evolut valves rendered coronary access more challenging and potentially unfeasible (24, 25). With the Evolut FX(+) valve, implantation can be optimized to facilitate coronary access by carefully tailoring the implant depth and achieving commissural alignment (17). The addition of three large windows with the FX+ further eases the challenge of coronary access, with each large cell size being four times larger than those in previous-generation Evolut systems (18).

**Figure 7:**
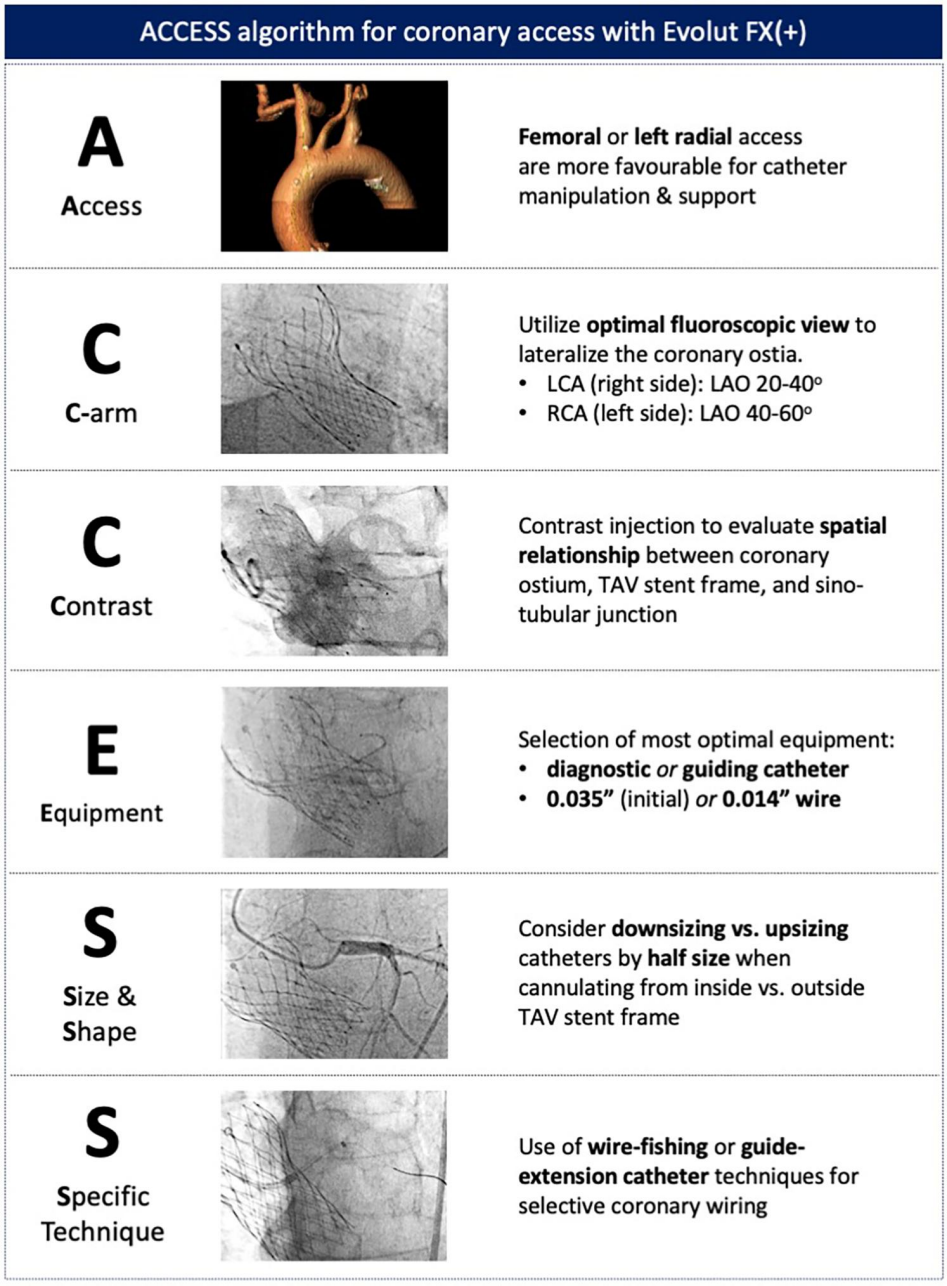
ACCESS algorithm for coronary access with the Evolut FX(+). A systematic approach to coronary access allows for potential challenges to be evaluated and specific techniques to be adopted to overcome coronary access challenges.

Despite these design improvements, coronary access may still be challenging and associated with adverse procedural outcomes, particularly during unplanned revascularization (26–28). For non-TAVI operators unfamiliar with valve design and cannulation techniques, the ACCESS algorithm provides a systematic approach to overcome the challenges associated with coronary access after TAVI (Figure 7). The first step involves considering either left radial or femoral access, which allows for the natural curvature of the aortic arch to be followed, providing increased catheter support and maneuverability. Second, the C-arm can then be utilized to evaluate the implanted TAV, determine its alignment, and obtain the optimal fluoroscopic view for the target coronary ostium. Third, a non-selective contrast injection or, if feasible, an aortogram can performed to evaluate the geometrical relationship between the TAV frame, aortic root, and coronary ostia. Particular attention should be given to the height of the coronary ostia and the valve-to-aorta (VTA) gap. Following this evaluation, a better understanding of the potential challenges faced during coronary cannulation can be determined, allowing for an appropriate selection of catheter size and shape. If initial attempts at selective cannulation are unsuccessful, specific techniques utilizing a 0.035-in. wire for further guide manipulation or a 0.014-in. to fish for the coronary ostium should be considered. During PCI procedures, the use of a guide-extension catheter is highly recommended, as it facilitates safe crossing of the TAV stent frame with balloons and stents.

## Conclusion

We describe how a dedicated implantation technique, utilizing the design modifications of the latest-generation Evolut FX(+), can help achieve an optimized index valve implantation. In addition, a systematic approach to commissural alignment and coronary access ensures that the Evolut FX(+) can be utilized as part of an effective lifetime management strategy for patients with severe AS.
